# Targeted temperature management in the ICU: guidelines from a French expert panel

**DOI:** 10.1186/s13613-017-0294-1

**Published:** 2017-06-19

**Authors:** Alain Cariou, Jean-François Payen, Karim Asehnoune, Gerard Audibert, Astrid Botte, Olivier Brissaud, Guillaume Debaty, Sandrine Deltour, Nicolas Deye, Nicolas Engrand, Gilles Francony, Stéphane Legriel, Bruno Levy, Philippe Meyer, Jean-Christophe Orban, Sylvain Renolleau, Bernard Vigue, Laure De Saint Blanquat, Cyrille Mathien, Lionel Velly, for the Société de Réanimation de Langue Française (SRLF) and the Société Française d’Anesthésie et de Réanimation (SFAR) In conjunction with the Association de Neuro Anesthésie Réanimation de Langue Française (ANARLF), the Groupe Francophone de Réanimation et Urgences Pédiatriques (GFRUP), the Société Française de Médecine d’Urgence (SFMU), and the Société Française Neuro-Vasculaire (SFNV)

**Affiliations:** 1 Medical Intensive Care Unit, Cochin University Hospital (APHP) & Paris Descartes University, 27 rue du Faubourg Saint-Jacques, 75014 Paris, France; 2 Pôle Anesthésie-Réanimation, Hôpital Michallon, CHU Grenoble Alpes, 38000 Grenoble, France; 3 Service de Réanimation médicale, CH Mulhouse, Mulhouse, France; 4 Hôpital Necker-Enfants Malades, Réanimation Pédiatrique Polyvalente, Assistance Publique - Hôpitaux de Paris, Paris, France; 5 Service d’Anesthésie-Réanimation, Hôpital de la Timone, Assistance Publique-Hôpitaux de Marseille, Marseille, France; 6 Hôpital Lariboisiere, Assistance Publique-Hôpitaux de Paris, Paris, France; 7 Centre Hospitalier de Versailles, Le Chesnay, France; 8 Hôpital Central, CHU de Nancy, Nancy, France; 9 CHU de Nancy, Nancy, France; 10 Fondation Rothschild, Paris, France; 11 Hôpital Pasteur 2, CHU de Nice, Nice, France; 12 Anesthésie-réanimation, Bicêtre, France; 13 CHU de Grenoble, La Tronche, France; 14 Hôpitial Hôtel-Dieu, CHU de Nantes, Nantes, France; 15 Hôpital Jeanne de Flandre, CHRU de Lille, Lille, France; 16 CHU de Bordeaux, Bordeaux, France; 17 Hôpital Necker-Enfants Malades, Assistance Publique-Hôpitaux de Paris, Paris, France; 18 Hôpital Armand-Trousseau, Assistance Publique-Hôpitaux de Paris, Paris, France; 19 Hôpital Pitié-Salpêtrière, Assistance Publique-Hôpitaux de Paris, Paris, France

## Abstract

Over the recent period, the use of induced hypothermia has gained an increasing interest for critically ill patients, in particular in brain-injured patients. The term “targeted temperature management” (TTM) has now emerged as the most appropriate when referring to interventions used to reach and maintain a specific level temperature for each individual. TTM may be used to prevent fever, to maintain normothermia, or to lower core temperature. This treatment is widely used in intensive care units, mostly as a primary neuroprotective method. Indications are, however, associated with variable levels of evidence based on inhomogeneous or even contradictory literature. Our aim was to conduct a systematic analysis of the published data in order to provide guidelines. We present herein recommendations for the use of TTM in adult and paediatric critically ill patients developed using the Grading of Recommendations Assessment, Development, and Evaluation (GRADE) method. These guidelines were conducted by a group of experts from the French Intensive Care Society (Société de Réanimation de Langue Française [SRLF]) and the French Society of Anesthesia and Intensive Care Medicine (Société Francaise d’Anesthésie Réanimation [SFAR]) with the participation of the French Emergency Medicine Association (Société Française de Médecine d’Urgence [SFMU]), the French Group for Pediatric Intensive Care and Emergencies (Groupe Francophone de Réanimation et Urgences Pédiatriques [GFRUP]), the French National Association of Neuro-Anesthesiology and Critical Care (Association Nationale de Neuro-Anesthésie Réanimation Française [ANARLF]), and the French Neurovascular Society (Société Française Neurovasculaire [SFNV]). Fifteen experts and two coordinators agreed to consider questions concerning TTM and its practical implementation in five clinical situations: cardiac arrest, traumatic brain injury, stroke, other brain injuries, and shock. This resulted in 30 recommendations: 3 recommendations were strong (Grade 1), 13 were weak (Grade 2), and 14 were experts’ opinions. After two rounds of rating and various amendments, a strong agreement from voting participants was obtained for all 30 (100%) recommendations, which are exposed in the present article.

## Introduction

The protective effects of hypothermia were first studied and described in the late 1950s and then seem to have been forgotten for roughly 20 years before intensivists revived interest in this therapeutic method [1, 2]. Experimental data show that the neuroprotective effects of therapeutic hypothermia occur through several mechanisms of action:

- reducing brain metabolism, which restores a favourable balance with cerebral blood flow in injured brain tissue [3, 4];
- lowering the intracranial pressure;
- reducing the initiation of brain cell apoptosis, notably the caspase activation pathway, and of necrosis [5];
- decreasing the local release of lactate and of excitotoxic compounds such as glutamate, that is associated with alteration in calcium homoeostasis during brain ischaemia [6–8];
- reducing brain tissue inflammatory response and systemic inflammatory response syndrome [7, 9];
- decreasing the production of free radicals [7, 10];
- limiting the vascular and cell membrane permeability as seen in brain ischaemia [10].

Accordingly, the use of mild to moderate hypothermia has gained interest for critically ill patients, in particular brain-injured patients in order to limit the extension of initial brain lesions [11]. This can be achieved through various methods and targeted temperatures. The term “targeted temperature management” (TTM) has emerged as the most appropriate referring to interventions used to reach and maintain a specific level temperature for each individual.

The level of targeted temperature may differ according to the situation. TTM may be used to prevent fever, to maintain normothermia, or to lower core temperature. TTM is widely used in intensive care units (ICUs) as a primary neuroprotective method, i.e. in order to protect against neuronal injury or degeneration in the central nervous system. Its indications are, however, associated with variable levels of evidence based on inhomogeneous or even contradictory literature. Our aim was to conduct a systematic analysis of the literature in order to edit national guidelines.

## Methods

These guidelines were conducted by a group of experts for the French Intensive Care Society (Société de Réanimation de Langue Française [SRLF]) and the French Society of Anesthesia and Intensive Care Medicine (Société Francaise d’Anesthésie Réanimation [SFAR]). The organization committee defined a list of questions to be addressed and designated experts in charge of each question. The questions were formulated using the PICO (Patient Intervention Comparison Outcome) format (http://www.gradeworkinggroup.org/).

The method used to elaborate these recommendations was the GRADE^®^ method [12]. Following a quantitative literature analysis, this method is used to separately determine the quality of available evidence, i.e. estimation of the confidence needed to analyse the effect of the quantitative intervention, and the level of recommendation. The quality of evidence is rated as follows:

-  high-quality evidence: further research is very unlikely to change the confidence in the estimate of the effect;
- moderate-quality evidence: further research is likely to have an impact on the confidence in the estimate of the effect and may change the estimate of the effect itself;
-  low-quality evidence: further research is very likely to have an impact on the confidence in the estimate of the effect and is likely to change the estimate of the effect itself;
-  very low-quality evidence: any estimate of the effect is very unlikely.

The level of recommendation is binary (either positive or negative) and strong or weak:

-  strong recommendation: we recommend (GRADE 1+) or we do not recommend to do (GRADE 1−);
-  weak recommendation: we suggest (GRADE 2+) or we do not suggest to do (GRADE 2−).

The strength of the recommendations is determined according to key factors and validated by the experts after a vote, using the Delphi and GRADE Grid method [13] that encompasses the following criteria:

-  the estimate of the effect;
-  the global level of evidence: the higher the level of evidence, the stronger the recommendation;
-  the balance between desirable and undesirable effects: the more favourable the balance, the stronger the recommendation;
-  values and preferences: in case of uncertainty or large variability, the level of evidence of the recommendation is probably weak, and values and preferences must be more clearly obtained from the affected persons (patient, physician, and decision-maker);
-  cost: the greater the costs or the use of resources, the weaker the recommendation.

The elaboration of a recommendation requires that at least 50% of voting participants have an opinion and that less than 20% of participants vote for the opposite proposition. The elaboration of a strong agreement requires the agreement of at least 70% of voting participants.

## Results

### Areas of recommendations

Fifteen experts and two coordinators agreed to consider questions concerning TTM and its practical implementation in five clinical situations in the intensive care setting: cardiac arrest (CA), traumatic brain injury (TBI), stroke, other brain injuries, and shock. The PubMed and Cochrane databases were searched for full articles written in English or French published after January 2005 and June 2015. In case of an absence or a very low number of publications during the considered period, the timing of publications could have been extended to 1995. The paediatric literature had a specific analysis.

The experts summarized the work and applied the GRADE^®^ method, resulting in 30 recommendations: 3 recommendations were strong (Grade 1), 13 were weak (Grade 2), and 14 were expert opinions. After two rounds of rating and various amendments, a strong agreement from voting participants was obtained for all 30 (100%) recommendations.

#### TTM after cardiac arrest

##### **R1.1**

We recommend using TTM in order to improve survival with good neurological outcome in patients resuscitated from out-of-hospital cardiac arrest (OHCA) with shockable cardiac rhythm (ventricular fibrillation and pulseless ventricular tachycardia) and who remain comatose after return of spontaneous circulation (ROSC).

**(Grade 1+)**

**Rationale:** Seven meta-analyses [14–20] and 4 systematic reviews [13, 21–23] assessed studies on targeted TTM after CA, but none identified separately patients with initial shockable versus non-shockable rhythm. Two studies with patients presenting initial shockable rhythm have been retained in the analysis [24, 25] and were in favour of the TTM use in this population. These trials (one pseudo-randomized [24] and one randomized [25]) found improved neurological outcome for OHCA patients with an initial shockable rhythm. Several studies were not included in the present analysis: (1) the TTM trial [26] because of the absence of a group with no control of body temperature, i.e. the trial compared TTM at 33 °C versus TTM at 36 °C, (2) a trial comparing a combination of hemofiltration with TTM versus hemofiltration alone versus no hemofiltration and no TTM [27], (3) clinical trials that assessed pre-hospital TTM. Observational studies, before–after studies, and matched cohort studies found results in favour of TTM use, despite a low to very low level of evidence [18]. International guidelines using the GRADE method and including Bernard [24] and HACA [25] trials strongly recommended the use of TTM for OHCA patients with an initial shockable rhythm to increase survival with good neurological outcome [13]. A recent meta-analysis also supports the use of TTM in this population [14].

##### **R1.2**

We suggest considering TTM in order to improve survival with good neurological outcome in patients resuscitated from OHCA with non-shockable cardiac rhythm (asystole or pulseless electrical activity) and who remain comatose after ROSC.

**(Grade 2+)**

**Rationale:** Among 8 meta-analyses [14–20, 28] and 4 reviews [13, 21–23] on TTM after CA, 1 meta-analysis specifically analysed patients with initial non-shockable rhythm [28]. This meta-analysis found decreased hospital mortality in patients with TTM: RR 0.86 (95% CI 0.76–0.99), but there was no difference regarding neurological outcome: RR 0.96 (95% CI 0.90–1.02). One randomized control trial [29] with a limited sample size (*n* = 30) compared patients treated with TTM *versus* no TTM and found no difference between the 2 groups. Most observational studies (low to very low level of evidence) did not observe significant between-groups differences. Although some studies were in favour of TTM use in this population [30–32], a majority of studies did not report a clinical improvement by using TTM [33–38]. Based on the poor prognosis in this population and considering the lack of therapeutic alternatives, the experts considered that TTM could be suggested in this population.

##### **R1.3**

We suggest considering TTM in order to increase survival with good neurological outcome in patients resuscitated from in-hospital cardiac arrest (IHCA) who remain comatose after ROSC.

**(Expert opinion)**

**Rationale:** There are no published randomized controlled trials with patients presenting in-hospital cardiac arrest (IHCA) patients. From 4 studies having included patients with IHCA or OHCA, it was not possible to separately analyse IHCA patients [37, 39–41]. In the overall population, the results are either in favour of TTM [31, 42] or neutral [34, 37, 39–41].

##### **R1.4**

We suggest considering TTM between 32 and 36 °C in order to improve survival with good neurological outcome after CA.

**(Grade 2+)**

**Rationale:** Two studies with a high level of evidence and consistent results addressed this question [14, 26]. One randomized controlled trial included 939 patients and found no significant difference in survival and neurological outcome between TTM at 33 °C versus TTM at 36 °C [26]. Similar results were reported in a meta-analysis [14]. This meta-analysis included 6 randomized studies using different degrees of temperature [24–27, 29, 43] and concluded that a TTM strategy using a targeted temperature <34 °C might improve outcome compared to a strategy with no TTM. A randomized controlled trial [43] comparing TTM at 32 versus 34 °C was not included in our analysis because this pilot trial included 36 patients only. A retrospective study, not retained in the analysis, found a better outcome at TTM 32–34 °C versus spontaneous normothermia below 37.5 °C [44]. *Post hoc* study derived from the TTM trial [26] was also not included to avoid duplicate data: it was found a possible worsening in outcome in the TTM 33 °C group for patients with shock at hospital admission [45]. Overall, targeted temperatures in clinical studies can be ranged between 32 and 36 °C, although it is not possible to define the optimal temperature within this range.

##### **R1.5**

We do not suggest initiating TTM with infusion of large volumes of cold saline solution during transportation to the hospital after CA.

**(Grade 2−)**

**Rationale:** Several randomized studies reported that pre-hospital infusion of cold fluids was not associated with improved outcome, whether this administration was conducted during resuscitation (intra-arrest cooling) or after ROSC [46–50]. In one randomized controlled trial [46], pre-hospital infusion of large volumes of cold saline solution (4 °C) was associated with increased occurrence of re-arrest after ROSC and of pulmonary oedema [46]. Alternative methods of pre-hospital cooling were too limited to assess impact on outcome [24, 51]. The impact of shortening time to reach TTM during the induction phase of TTM is discussed elsewhere (R6.1). Conversely, any onset of hyperthermia after ROSC appears deleterious in terms of outcome [52, 53].

##### **R1.1**

Paediatric—In comatose children following resuscitation from OHCA or IHCA with a shockable or a non-shockable cardiac rhythm, we recommend using TTM to maintain normothermia in order to improve neurological outcome.

**(Expert opinion)**

**R1.2** Paediatric—in comatose children following resuscitation from OHCA or IHCA with a shockable or a non-shockable cardiac rhythm, we do not suggest using TTM between 32 and 34 °C to improve survival with good neurological outcome.

**(Grade 2−)**

**Rationale**: In a randomized study [54], 270 children (2 days–18 years) with OHCA were compared: 142 children allocated to TTM at 33 °C versus 128 to TTM 36–37.5 °C during 5 days. There was no significant difference between groups in survival rate with good neurological outcome. In this population, hypoxia was the main cause of OHCA (72%), which may explain why these results differed from adults [24, 25]. In a recent study, TTM lower than 32 °C was associated with higher mortality rate [55]. Other retrospective or prospective studies had limited population size, and mixed OHCA and IHCA, cardiac and respiratory causes. Although hypothermia was still recommended in 2010 [56, 57], the latest recommendations by the International Liaison Committee on Resuscitation (ILCOR) endorsed the latest trials and did not recommend any longer using hypothermia in children who remain comatose after resuscitation CA [58].

#### TTM after traumatic brain injury

##### **R2.1**

In patients with severe traumatic brain injury, we suggest considering TTM at 35–37 °C in order to control intracranial pressure.

**(Grade 2+)**

**Rationale:** In a case–control series of patients with severe traumatic brain injury (TBI), Puccio et al. [59] showed that patients with TTM maintained at 36–36.5 °C within 72 h of TBI presented a lower averaged intracranial pressure (ICP) and fewer episodes of ICP >25 mmHg as compared with patients who did not receive TTM. Several series of clinical cases [60–62] showed a correlation between core temperature, brain temperature, and ICP. However, there is no randomized controlled study showing that TTM at 35–37 °C in patients with severe TBI was associated with prevention of intracranial hypertension.

##### **R2.2**

In patients with severe traumatic brain injury, we suggest considering TTM at 35–37 °C to improve survival with good neurological outcome.

**(Grade 2+)**

**Rationale:** In patients with TBI, hyperthermia is associated with higher mortality rates [63, 64], unfavourable neurological outcome [63–66], and prolonged length of stay in the ICU and in the hospital [63, 67, 68]. A cohort of patients with head injury (65% of TBI) did not show improved neurological outcome in patients who received TTM as compared with historical controls with no TTM [69]. There is yet no randomized controlled study showing that TTM in patients with TBI is associated with improved neurological outcome, reduced mortality rate, or length of hospital stay. Despite the low level of evidence, the aggravated outcome associated with hyperthermia supports the recommendation to maintain TTM between 35 and 37 °C.

Several studies with a higher level of evidence [70–74] and meta-analyses [75–77] in adults and children showed no benefit regarding mortality or neurological outcome with the use of therapeutic hypothermia between 32 and 35 °C, compared to normothermia in patients with severe TBI whether they had intracranial hypertension or not.

##### **R2.3**

In TBI patients with refractory intracranial hypertension despite medical treatments, we suggest considering TTM at 34–35 °C in order to lower ICP.

**(Grade 2+)**

**Rationale:** Several studies showed that TTM at 34–35 °C could lower ICP [70, 75, 78–84], while this effect was not confirmed elsewhere [71, 72], and a raised in ICP was seen during rewarming [69]. Polderman et al. [85] showed in 136 patients (64 patients receiving barbiturates + hypothermia versus 72 patients receiving barbiturates alone) that therapeutic hypothermia was superior to barbiturates alone to treat high ICP and was associated with a reduced mortality rate. In the Eurotherm trial [86], which is the first randomized, multicentre study in a population of TBI patients with ICP above 20 mmHg, 195 patients were treated with TTM (32–35 °C) versus 192 patients treated with normothermia. High ICP was easier to control in patients of the TTM group (barbiturates were used in 41 control patients versus 20 TTM patients). However, several studies found no additional clinical benefit where lowering temperature below 34 °C. In 22 patients with severe head injury, Shiozaki et al. [87] found that intracranial hypertension persisting at 34 °C was not reduced any further by lowering the temperature up to 31 °C. Using multimodal monitoring, hypothermia below 35 °C showed no benefit regarding brain tissue oxygen tension (PtiO2) or biomarkers of brain metabolism [60], and this strategy has a deleterious effect on these parameters [88]. TTM between 32 and 35 °C to treat high ICP can result in side effects, e.g. respiratory infections, that are proportional to the duration and/or depth of hypothermia [75, 76].

The duration of hypothermia should be adapted according to the persistence of intracranial hypertension. In a study of 215 patients with severe TBI, a 5-day duration of hypothermia resulted in better control of ICP and neurological outcome than a 2-day period. In addition, rewarming after 5 days resulted in less rebound effect of ICH than after 2 days [89]. The meta-analysis of McIntyre et al. [90] was in line with these findings.

Sustained elevation of ICP is an independent factor of poor outcome after TBI. Uncontrolled studies indicated that therapeutic hypothermia to treat high ICP could be associated with better outcome [77, 78, 82, 91]. However, the Eurotherm study [86] showed that TTM had a negative effect on neurological outcome at 6 months with an odds ratio of unfavourable outcome after adjustment at 1.53 (1.02–2.30) (*P* = 0.04); favourable outcome (Glasgow Outcome Scale Extended score 5–8) 26% in the TTM 32–35 group versus 37% for patients in the control group (*P* = 0.03). It should be noted that ICP was moderately elevated in that trial, i.e. 20–30 mmHg and of short-term duration (5 min), and that therapeutic hypothermia was initiated with cold saline infusion prior to using treatments for high ICP (osmotic therapy).

##### **R2.1**

Paediatric—In children with severe traumatic brain injury, we recommend using TTM at normothermia.

**(Expert opinion)**

**R2.2** Paediatric—In children with severe traumatic brain injury, we do not recommend using TTM at 32–34 °C to improve outcome or control intracranial hypertension.

**(Grade 1−)**

**Rationale:** A randomized study including 225 patients [70] and another one with 77 patients, which was terminated prematurely because of futility [73], support the conclusion that moderate hypothermia (32–34 °C) provides no benefit in terms of outcome in severely brain-injured children. A post hoc study by Hutchison et al. [92] showed a higher incidence of low arterial blood pressure and low cerebral perfusion pressure during moderate hypothermia (32–34 °C). A retrospective study in 117 severe TBI children identified early hyperthermia as an independent factor associated with aggravated outcome (OR for lower Glasgow Coma Scale score at PICU discharge: 4.7, CI 1.4–15.6; OR for longer length of stay in PICU: 8.5, CI 2.8–25.6) [93]. TTM may reduce ICP in TBI children as seen in adults.

#### TTM after stroke, intra-cerebral haemorrhage, and subarachnoid haemorrhage

##### **R3.1**

We suggest considering TTM at normothermia during the early phase of severe ischaemic stroke.

**(Expert opinion)**

**Rationale:** Hyperthermia or fever is a frequent complication (>50%) in patients at the acute phase of stroke and is correlated with poor functional outcome [63]. However, the efficacy of therapeutic hypothermia has not yet been shown, according to 6 randomized trials that tested hypothermia (33–35 °C) in stroke patients [94–99]. There were few patients and methodological biases were numerous. A single study [99] investigated patients with severe stroke (NIHSS >15). Two randomized studies are ongoing: EuroHYP-1 [100] explores the value of 24 h; 34–35 °C hypothermia following recent stroke, and the recent ICTuS 2/3 trial [101]. In that later study, intravascular therapeutic hypothermia was found safe and feasible in patients treated with recombinant tissue-type plasminogen activator.

Routine use of antipyretics is commonly recommended in patients with hyperthermia, though there is no evidence of their impact on neurological outcome or mortality. The meta-analysis of Ntaios et al. [102] that included 4 major randomized trials [103–106] found no difference in mortality or neurological outcome between hypothermia and hyperthermia (>38 °C).

##### **R3.2**

In comatose patients with spontaneous intra-cerebral haemorrhage, we suggest considering TTM at 35–37 °C to lower intracranial pressure.

**(Expert opinion)**

**Rationale:** Observational studies showed that fever is indicative of poor neurological outcome after intra-cerebral haemorrhage [107, 108]. However, most of these studies were observational, with a small number of patients and methodological biases. Two observational studies showed that hypothermia at 35 °C over 8–10 days had a favourable effect on peri-haemorrhagic oedema and ICP, with no benefit on neurological outcome [109, 110]. A case–control study using TTM at 37 °C found no effect on neurological outcome at ICU discharge, while the duration of mechanical ventilation and length of stay in the ICU was prolonged in patients treated with TTM [111]. The effect of hypothermia at 35 °C during 8 days is ongoing in patients with a large intra-cerebral haemorrhage (25–64 mL) (CINCH trial) [112].

##### **R3.3**

In comatose patients with aneurysmal subarachnoid haemorrhage, we suggest considering TTM to lower ICP and/or to improve neurological outcome.

**(Expert opinion)**

**Rationale:** Observational studies showed that fever is predictive of poor neurological outcome after subarachnoid haemorrhage [113–115]. Most of these studies were observational, with a small number of patients and methodological biases [116–120]. These studies found a decrease in ICP and suggested that the 12-month neurological outcome might be improved with the use of normothermia and hypothermia (32–34 °C) in refractory intracranial hypertension. A randomized controlled study [118] compared normothermia (TTM at 36.5 °C) versus conventional treatment for hyperthermia >37.9 °C: no conclusion could be drawn for the subgroup of patients with subarachnoid haemorrhage.

##### **R.3.1**

Paediatric 3.1 Paediatric—In children with subarachnoid haemorrhage, we suggest considering TTM between 36 and 37.5 °C to control intracranial pressure.

**(Experts’ recommendation)**

**Rationale**: There is no randomized controlled study that explored the use of TTM and therapeutic hypothermia in children with stroke, intra-cerebral haemorrhage, and/or subarachnoid haemorrhage. Reduction of intracranial pressure was reported in one clinical case [121] and in a retrospective study where fever control and therapeutic hypothermia were used [122]. TTM may reduce ICP in children with SAH as seen in adults.

#### TTM in acute bacterial meningitis and status epilepticus

##### **R4.1**

In patients with refractory or super-refractory status epilepticus, we suggest considering TTM at 32–35 °C to control seizure activity.

**(Expert opinion)**

**Rationale:** Experimental studies [123–131] showed the anticonvulsant properties of hypothermia that were confirmed in patients with refractory or super-refractory status epilepticus (lasting for more than 24 h) persisting despite maximum treatment. A randomized controlled trial and several reports showed that TTM (32–35 °C) for 24 h was associated with a better control of electrical seizure activity and achievement of burst-suppression pattern [132–134]. In the HYBERNATUS trial, the rate of progression to EEG-confirmed status epilepticus was lower in the hypothermia group than in the control group (11 vs. 22%; odds ratio, 0.40; 95% CI 0.20–0.79; *P* = 0.009) [135].

##### **R4.2**

In comatose patients with meningitis or meningoencephalitis, we do not suggest considering TTM when fever is tolerated.

**(Expert opinion)**

**Rationale:** No interventional study tested the effect of TTM on outcome of ICU patients with meningitis or meningoencephalitis. Saxena et al. [136] showed that peak temperature in the first 24 h of ICU admission did not increase the hospital mortality rate in patients with infection of the central nervous system (CNS), whereas it was associated with increased mortality rate in patients with stroke and TBI. Other studies did not report consistent results [137, 138]. Interestingly, in the absence of infection, the outcome was better if the temperature over the first 24 h peaked between 37.5 and 37.9 °C, whereas in case of infection the outcome was better if temperature peaked at 38–38.4 °C (UK database) or at 39–39.4 °C (NZ/Australian database) [136]. Fever plays a protective role because it inhibits replication of *N. meningitidis* and *S. pneumoniae* [136] and eases the diagnosis of infection [137]. The use of paracetamol in septic patients can decrease temperature by 0.3 °C, but did not affect mortality or length of ICU stay [139].

##### **R4.3**

In comatose patients with bacterial meningitis and no intracranial hypertension, we suggest considering normothermia to improve survival and neurological outcome.

**(Expert opinion)**

**Rationale:** In ICU patients with bacterial meningitis, Mourvillier et al. [140] found more deleterious effects in the induced hypothermia group. No other study has been conducted on this topic.

##### **R4.4**

In comatose patients with bacterial meningitis and intracranial hypertension, we suggest considering TTM at 34–36 °C to improve survival and neurological outcome.

**(Expert opinion)**

**Rationale:** In comatose patients with bacterial meningitis and intracranial hypertension, hypothermia had a favourable effect on non-invasive measurements of ICP [141]. However, the control group was historical and 10 patients from a preliminary study were probably included [142]. In a case report [143], TTM at 35–36 °C controlled refractory intracranial hypertension, in combination with thiopental. In two other studies in patients with severe viral encephalitis and intracranial hypertension, outcome was better in cooled patients [144, 145]. Similar findings were obtained in other case reports [146–151].

##### **R4.1**

Paediatric—In children with status epilepticus, we suggest considering TTM (normothermia) to improve outcome.

**(Expert opinion)**

**Rationale**: Few clinical cases of refractory or super-refractory status epilepticus [152–154] and encephalitis in children [152, 155] treated with therapeutic hypothermia have been reported. There is a lack of evidence to formalize recommendation. A retrospective study in 43 heterogeneous patients (encephalitis and encephalopathy—viruses, major hyperthermia, shock, status epilepticus) [156] compared 16 patients under normothermia versus 27 patients under therapeutic hypothermia. The cerebral performance category score at 12 months was better in patients under hypothermia. Maintaining normothermia in children with status epilepticus may be appropriate.

#### TTM after haemodynamic shock

##### **R5.1**

We do not suggest considering TTM below 36 °C in patients with cardiogenic shock.

**(Grade 2−)**

**Rationale:** There were 4 studies that investigated the effects of moderate hypothermia [157–160]. They were retrospective or uncontrolled, and the number of patients included was low. Therapeutic hypothermia is feasible and not associated with an increased incidence of adverse effects. A prospective study is in progress (clinical trial.gov—NCT01890317).

##### **R5.2**

We do not suggest considering TTM below 36 °C in patients with septic shock.

**(Grade 2−)**

**R5.3** We suggest considering TTM at normothermia in patients with septic shock.

**(Grade 2)**

**Rationale:** Among five randomized trials [161–164], three were designed to evaluate non-steroidal anti-inflammatory drugs versus placebo with a large proportion of patients without fever in the 2 groups [161, 162, 164]. One study compared acetaminophen versus placebo in patients with fever [139]. These studies showed that antipyretics effectively control temperature with no reported side effects. There was no difference in mortality rate, hemodynamic status, or length of stay in ICU. In one trial that compared TTM versus no TTM, Schortgen et al. [163] found that the vasopressor support (main endpoint) was significantly reduced in the TTM group, as was the duration of shock. However, despite a decreased mortality rate (secondary endpoint) at day 14 observed in the TTM group, there was no difference in mortality rate at both ICU and hospital discharge. TTM improved hemodynamic status in one methodologically well-conducted study [163] in which mortality was a secondary objective. All clinical studies concluded that TTM is feasible in septic shock and is not associated with more frequent side effects.

#### Implementation and monitoring of TTM

##### **R6.1**

We recommend using advanced methods with servo-regulated cooling of central body temperature to optimize TTM.

**(Grade 1+)**

**Rationale:** The present recommendation is based on post-cardiac arrest patients. A greater efficacy of advanced methods (“servo-controlled”) was constantly found, particularly during the maintenance phase. The benefit of advanced methods during the induction phase of TTM was however variable because it relied upon the initial temperature. The incidence of overcooling/overshooting was variable between advanced and basic methods. Our analysis included 3 randomized studies [165–167] and uncontrolled studies [168–171]. Regarding the neurological outcome, results were discordant: one study found a trend in favour of advanced methods [165], two studies were either negative [166] or positive (though cerebral performance category score was not reported) [167], and 4 studies gave variable results [168–171]. Overall, these advanced methods might help to optimize TTM, but their effect on survival with good neurological outcome was not established [40].

##### **R6.2**

We suggest considering the control of rewarming in patients treated with TTM.

**(Expert opinion)**

**Rationale:** After cardiac arrest, it is impossible to distinguish effects associated with the rewarming period from those attributable to induction and maintenance phase. Experts retained 2 randomized trials [165, 166] that found no differences between controlled and passive rewarming. The rate of rewarming and the time to achieve normothermia were found difference between controlled and uncontrolled rewarming in non-randomized studies [178–180]. Rebound or post-rewarming fever was not always suppressed using controlled rewarming [165, 170–172]. Clinical studies found no association between rewarming rate and outcome after adjustment [165, 171–174]. In brain trauma and stroke, a lower rate of rewarming is important in order to prevent rebounds on ICP [89, 173, 174]. However, no randomized trial has specifically addressed this issue other than indirect measurements of ICP [175].

##### **R6.3**

We suggest considering measurements of core body temperature in patients treated with TTM.

**(Grade 2+)**

**Rationale:** Besides the brain as the most preferable site to measure core body temperature, other sites can be used. Observational studies assessed the agreement between the core body temperature and other sites, i.e. oesophagus, bladder, rectum, nasopharynx, eardrum, and skin [176–180]. Agreement between core body sites of measurement (lungs, oesophagus, bladder, and rectum) was correct. Correlation and agreement were poor for peripheral sites of measurement (skin and tympanic) [176–180]. A study reported a bias of 1 °C between core and tympanic temperature [178].

##### **R6.4**

We suggest considering the detection of several complications (sepsis, pneumonia, arrhythmia, hypokalaemia) in patients treated with TTM.

**(Grade 2+)**

**Rationale:** The meta-analysis of Xiao et al. [181] found a trend between the use of TTM and the incidence of infectious complications, i.e. sepsis and pneumonia. Not included in this meta-analysis are observational studies performed in large populations [33, 182–184]: they all found an increased incidence of infectious complications in TTM patients.

Hypokalaemia is the most common metabolic derangement reported in TTM studies [26]. Two studies reported an increased occurrence of hypokalaemia (serum potassium <3.5 mmol/L) [37, 185] that was confirmed in the meta-analysis of Xiao et al. [181]. This meta-analysis found higher risk of cardiac arrhythmia in patients treated with TTM [181].

##### **R6.1**

Paediatric—We suggest considering methods with servo-regulated cooling of central body temperature in children treated with TTM.

**(Expert opinion)**

**Rationale**: One randomized open controlled study showed the superiority of a servo-controlled system in maintaining hypothermia in newborns with perinatal asphyxia managed in the pre-hospital settings with therapeutic hypothermia [186]. Forty-nine infants were allocated to a servo-controlled system, and 51 were cooled according to the unit’s usual protocol (passive hypothermia). Newborns cooled with the servo-controlled device were more often close to the target temperature upon arrival at the hospital (median 73% [IQR 17–88] vs. 0% [IQR 0–52] *P* < 0.001). The target temperature was more often reached during transport in the servo-control group (80 vs. 49%, *P* < 0.001) and in a shorter time (44 ± 31 vs. 63 ± 37 min, *P* = 0.04). The number of newborns who reached the target temperature in 1 h was significantly higher in the servo-control group than in the control group (71 vs. 20%, *P* < 0.001).

##### **R6.2**

Paediatric—We suggest considering measurements of core body temperature measurement in children treated with TTM.

**(Grade 2+)**

**Rationale**: Several studies compared the accuracy of different sites for temperature measurement in children. In a prospective study (*n* = 15), peripheral sites (axillary, tympanic, rectal, oesophageal) were compared to central blood measurements [187]. The best agreement with blood temperature was obtained with oesophageal measurements. Several other studies and meta-analysis confirmed a poor agreement between axillary or tympanic measurements and central blood temperature [188–190].
